# Bioaerosols of subterraneotherapy chambers at salt mine health resort

**DOI:** 10.1007/s10453-013-9298-y

**Published:** 2013-03-16

**Authors:** Krzysztof Frączek, Rafał L. Górny, Dariusz Ropek

**Affiliations:** 1 Department of Microbiology, University of Agriculture, 24/28 Mickiewicza Avenue, 30-059 Kraków, Poland; 2 Biohazard Laboratory, Department of Chemical, Aerosol and Biological Hazards, Central Institute for Labour Protection - National Research Institute, 16 Czerniakowska Street, 00-701 Warsaw, Poland; 3 Department of Agricultural Environment Protection, University of Agriculture, 21 Mickiewicza Avenue, 31-120 Kraków, Poland

**Keywords:** Air quality, Bioaerosol, Salt mine, Health resort, Subterraneotherapy

## Abstract

Nowadays, an inhalation of naturally generated aerosols has again become a widely practiced method of balneological treatment of various respiratory diseases. The aim of this study was to characterize the microbial aerosol of subterraneotherapy chambers at the Bochnia Salt Mine Health Resort in southern Poland. The measurements were carried out using a 6-stage Andersen impactor over a period of 1 year in both indoor (i.e., two subterranean chambers, where curative treatments took place) and outdoor air. The maximum bacterial aerosol concentrations in the chambers reached 11,688 cfu/m^3^. In such interiors, a high-performance method of microbial contaminant reduction need be introduced, especially when large groups of young patients are medically cured. Respecting fungal aerosol, its average indoor concentration (88 cfu/m^3^) was significantly lower than outdoor level (538 cfu/m^3^). It confirms that ventilation system provides efficient barrier against this type of biologically active propagules. Among identified micro-organisms, the most prevalent indoors were Gram-positive cocci, which constituted up to 80 % of airborne microflora. As highly adapted to the diverse environments of its human host (skin, respiratory tract), they can be easily released in high quantities into the air. The number of people introduced into such subterranean chambers should be in some way limited. The analysis of microclimate parameters revealed that temperature and relative humidity influenced significantly the level of bacterial aerosol only. Hence, a constant control of these parameters should be scrupulously superintended at this type of subterranean premises.

## Introduction

The twenty-first century has been called “the age of prevention” as more and more people have been consciously taking care of their health. In this context, numerous natural therapy methods have again become a widely practiced in medical treatment of different diseases. One of the flagship examples of this type is balneology. This method is based on naturally (or artificially) formed microclimate conditions in the subterranean chambers. Despite the known benefits of such treatments, a subterraneotherapy is implemented in a few health resorts in the world only (e.g., in Berchtesgaden in Germany, Solotwino in Ukraine, Zlaté Hory in Czech Republic or Wieliczka and Bochnia in Poland). According to the Latin maxim “*medicus curat*, *natura sanat*” (i.e., “*medicine cures*, *nature heals*”), in the 1950s, a Polish professor Mieczysław Skulimowski and German doctor Karl Hermann Spannagel created a modern scientific and medical basis for this type of treatment. In 1958 in Wieliczka, nearby Cracow, Poland, the world’s first salt spa for patients with respiratory diseases was open. The unique presence of dry salt aerosols having both specific concentration and size maintains “clean,” that is, micro-organism- and allergen-free atmosphere. Such elimination and/or attenuation of those immunologically reactive stimulants determines the efficiency of subterraneotherapy and in numerous cases may heal adverse health effects (Bihari-Axelsson and Axelsson 2002; Chervinskaya 2007; Fiegel et al. 2006; Helben and Kolarzyk 2005; Kmiecik 2006, 2007; Olechnowicz-Bobrowska and Wojkowski 2004; Szczegielniak and Migała 2003). The Bochnia Salt Mine Health Resort specializes in medical treatment of respiratory tract diseases (such as recurrent infections of the upper and lower airways, allergic and chronic non-allergic rhinitis, chronic inflammation of the pharynx and larynx, asthma, chronic bronchitis and bronchiectasis) using a method under which special breathing and gymnastic exercises are carried out in the unique microclimate of the subterranean chambers.

There is broad medical evidence that specific microclimate conditions in subterranean chambers (or artificially created salt chambers) have a beneficial effect on human health, especially in case of respiratory diseases. The importance of this phenomenon is emphasized by the fact that current environmental changes, particularly the increase in gaseous and particulate pollutant concentrations in the air, reduce the natural immunity of human organism (Anderson et al. 1997; Chervinskaya 2003, 2007; Doleżal et al. 1983; Garavello et al. 2003; Kmiecik 2007). Pulmonological studies revealed that an immediate effect of subterraneotherapy is usually visible in about 15 % of patients and very good or good direct effect after termination of the treatment cycle is observed in about 90 % of them. Long-term effect (from 3 to several months from the first treatment) may be also observed in about 60 % of patients (Bihari-Axelsson and Axelsson 2002; Bis et al. 2004; Chervinskaya 2003; Chervinskaya and Zilber 1995; Frączek et al. 2008); however, the effectiveness of such treatment has certain limitations and is usually inversely proportional to the age of the patient, duration of the disease, subsequent pathological changes and concomitant disorders. Poor or no effect is more pronounced in elderly patients, among whom the course of respiratory diseases is prolonged with severe subsequent changes and concomitant illnesses (Chervinskaya 2007; Kmiecik 2007; Skulimowski 1964).

As microbial quality of the air is a key factor in inhalation therapy, the aim of this study was to quantitatively and qualitatively characterize bacterial and fungal aerosols in subterranean chambers of the Bochnia Salt Mine Health Resort (the oldest salt mine in Poland). Both an impact of microclimate parameters (i.e., temperature and relative humidity) as well as an influence of atmospheric air (forced mechanically into the analyzed chambers) on the indoor microbial population were also evaluated.

## Materials and methods

The study was carried out in the Bochnia Salt Mine Health Resort, being a part of the oldest salt mines in Poland. The Bochnia Salt Mine origin dates back to the thirteenth century, and it possesses the oldest functioning shaft in European mining constructed in 1251. Since 1995, a few mine excavations have been converted into a sanatorium and used for therapeutic purposes (mainly inhalation and rehabilitation stays).

The bioaerosol measurements were performed over a period of 1 year (from January to December), twice in each of four seasons. The air samples were collected in two subterranean chambers: Ważyn (255 m long, 11,450 m^3^, the Europe’s biggest man-made subterranean room) and Kołdras (1,110 m^3^), located 248 and 176 m below the ground level and housing 250 and 42 beds for patients, respectively. In both chambers, the samples were taken during the treatment courses with patients and in the same premises 24 h after curative treatment without them. This 24 h break before the post-treatment sampling was chosen due to the scheme of exchange between the “old” (i.e., already treated) and “new” (i.e., before treatment) groups of patients. The patient groups (150–200 people) consisted mostly of children. Majority of the patients (90 %) recruited from among elementary school children; their parents, teachers or chaperons made the remaining 10 % of the persons present in the chambers. Sampling was carried out 24 h after curative treatment of patients in empty chambers. Moreover, the air samples were taken from the ramp, an incline of 139 m long being a part of the corridor connecting Ważyn and Kołdras chambers. In addition, in each of the investigated areas, an indoor background station was established to measure bacterial and fungal aerosol concentrations (IN-B). All these measurements were carried out in the galleries located on the same levels as the studied chambers, through which the air was introduced into them.

**Fig. 1:**
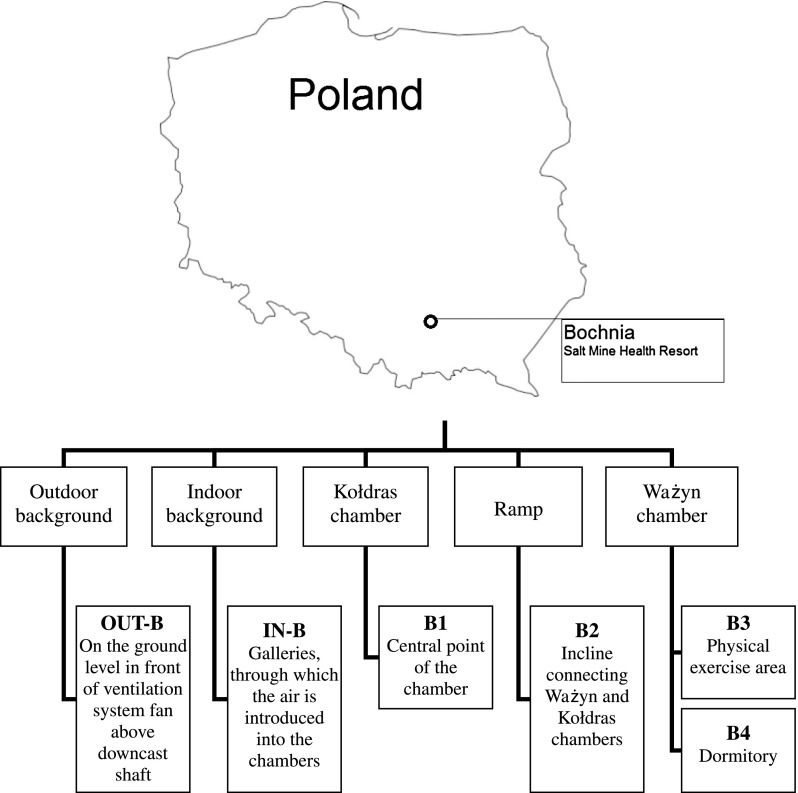
Location of the Bochnia Salt Mine Health Resort and short characteristics of sampling points

The mechanical ventilation system placed above the ground and equipped with a high-performance fan (WLE—1004A/1, POWEN Zabrze), and filter set (cotton filter) was responsible for a delivery of the atmospheric air into the studied subterranean chambers. The air was forced down into the chambers from the surface level through an old (and not cleaned recently) 210 m long brick downcast shaft (housing a wooden ladder way), and then its movement in the underground spaces was regulated by the barrier walls and a specific location of therapy chambers in the corridor bends. The shaft was unheated. In order to obtain data on the background atmospheric level of microbial contamination (OUT-B), the air was sampled in front of a fan outside the salt mine above the ground. The location of the Bochnia Salt Mine Health Resort and selected sampling points characteristics is presented in Fig. 1.

The air samples were collected using a 6-stage Andersen impactor (model 10-710, Graseby-Andersen, Inc., Atlanta, GA, USA). The sampler was placed at a height of 1.0–1.5 m above the floor or ground level to simulate aspiration from the human breathing zone. A 5 min sampling period was applied for collection of bacterial and fungal aerosols. Samples were taken at a flow rate of 28.3 L/min. All bioaerosol samples were taken in triplicates. Bacteria were collected, on blood trypticase soy agar (TSA; Becton, Dickinson and Company, Sparks, MD, USA), and after impactor reloading, fungi were collected on malt extract agar (MEA; Oxoid Ltd., Basingstoke, Hampshire, Great Britain). During sampling, the air temperature and relative humidity (RH) were measured using a hytherograph (model Omniport 20, E + E Elektronik Ges.m.b.H., Engerwitzdorf, Austria).

After sampling, the TSA plates were incubated for 1 day at 37 °C followed by 3 days at 22 °C and another 3 days at 4 °C, and MEA plates for 4 days at 30 °C followed by 4 days at 22 °C (Dutkiewicz 1978). After incubation of the plates, the qualitative and quantitative analyzes of growing micro-organisms were performed. The concentration of bioaerosols was calculated as colony forming units per cubic meter of the air (cfu/m^3^). Bacterial strains were identified to the genus and/or species level by Gram staining, their morphology and, finally, by the biochemical API tests (bioMérieux, Marcy l’Etoile, France). Fungi were identified according to their morphology using several identification keys (Atlas 2004; Domsch et al. 1980; Fassatiowa 1983; Raper and Fennel 1965; Samson et al. 2004).

As the collected data had a nonparametric distribution, the statistical analyzes were performed by Kruskal–Wallis (to compare three or more groups of data, for example, differences between the seasons) and Mann–Whitney tests (to compare two independent samples, for example, bioaerosol concentrations in chambers with and without patients) as well as Spearman correlation (to find the relationships between bioaerosol concentrations and microclimate parameter values) using Statistica (data analysis software system) version 7.1—2006 (StatSoft, Inc., Tulsa, OK, USA). The “*p*” values lower than 0.05 were treated as statistically significant.

## Results

**Fig. 2:**
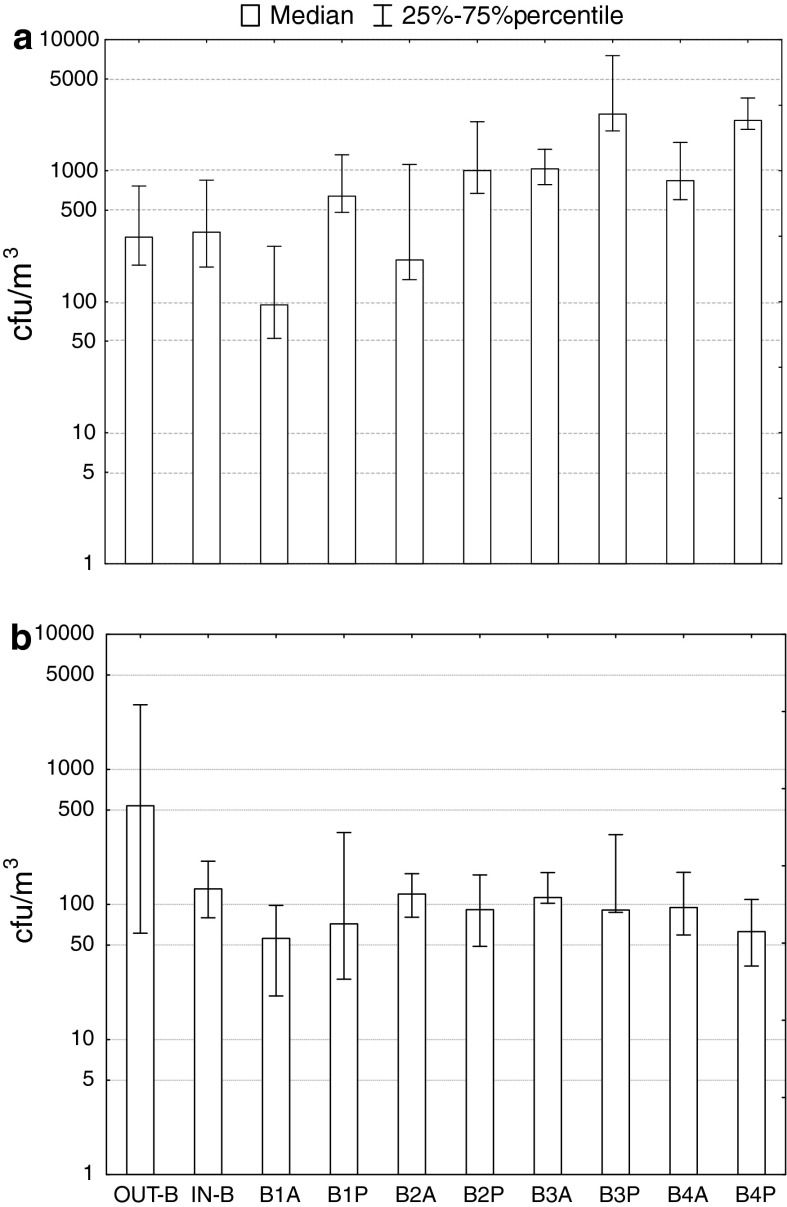
Average concentrations (cfu/m^3^) of bacteria (**a**) and fungi (**b**) in outdoor and indoor air at the Bochnia Salt Mine Health Resort. The *symbols* represent: *OUT*-*B* outdoor background, *IN*-*B* indoor background, *B1* the Kołdras chamber, *B2* Ramp, *B3* the Ważyn chamber: physical exercise area, *B4* the Ważyn chamber: dormitory, *A* absence of patients, *P* presence of patients

**Fig. 3:**
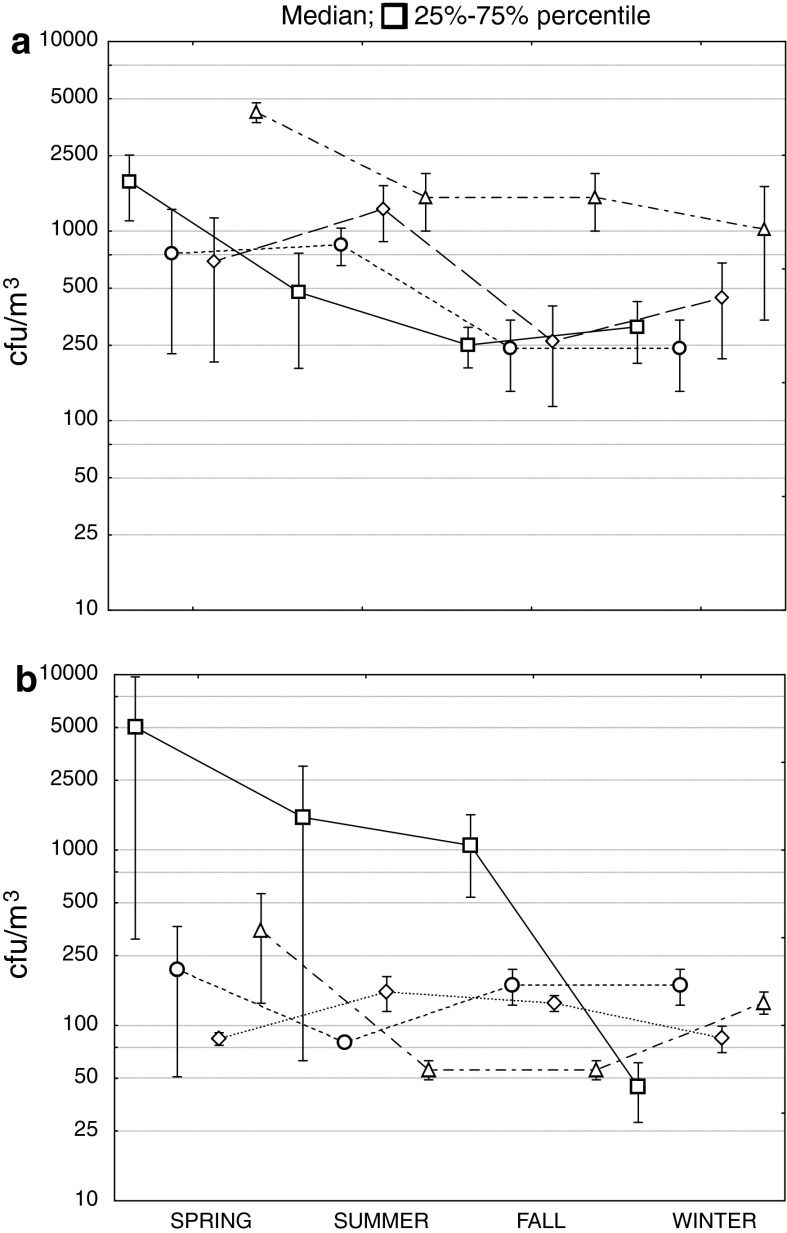
Seasonal variations in bacterial (**a**) and fungal (**b**) aerosol concentrations (cfu/m^3^). The *symbols* represent: *square* outdoor background, *circle* indoor background, *diamond* premises without patients and *triangle* premises with patients

The bioaerosol concentrations in subterraneotherapy chambers at the Bochnia Salt Mine Health Resort are presented in Table 1 and Fig. 2. Bacterial aerosol concentrations ranged from 35 to 11,688 cfu/m^3^ and from 189 to 2,517 cfu/m^3^ in indoor and outdoor air, respectively. The highest bacterial aerosol concentrations were observed in the Ważyn chamber (11,688 cfu/m^3^) during physical activity of the patients. When the patients were absent, the measured bacterial concentrations in the air were significantly lower than when they were present (median values: 2,010 versus 545 cfu/m^3^; Mann–Whitney test: *p* < 0.05), reaching the level characteristic for both indoor and outdoor backgrounds. There were no statistically significant differences between the bacterial aerosol concentrations in the outdoor background, indoor background and subterranean chambers after curative treatment without patients (Kruskal–Wallis test: *p* > 0.05). The comparison of bacterial aerosol concentrations between the studied premises revealed the highest differences (Kruskal–Wallis test: *p* < 0.05) between the physical exercise area in the Ważyn chamber (median: 2,948 cfu/m^3^) and the Kołdras chamber (median: 530 cfu/m^3^; Fig. 2a). The analysis of seasonal changes in bacterial aerosol concentrations in the premises with and without patients revealed a significant difference between spring and fall (Kruskal–Wallis test: *p* < 0.05; Fig. 3a).

**Table 1:** Bacterial and fungal aerosol concentrations (cfu/m^3^) of the air at the Bochnia Salt Mine Health Resort

Environment	Bacteria		Fungi	
	Range	Median	Range	Median
Subterranean chambers				
During treatment courses with patients	322–11,688	2,010	7–566	88
After curative treatment without patients	35–2,409	545	7–226	102
Indoor background	143–1,302	339	51–367	131
Outdoor background	189–2,517	311	28–9,731	538

Regarding fungal aerosol, its concentrations in the studied premises (with and without patients as well as in indoor background) ranged from 7 to 566 cfu/m^3^ (Table 1). Outdoor fungal aerosol levels were able to reach 9,731 cfu/m^3^ and were significantly higher than those observed indoors (Mann–Whitney test: *p* < 0.05). Moreover, it was shown that the concentrations of fungi in chambers during treatment courses (i.e., in the presence of staff and patients) and without them were not statistically different (Mann–Whitney test: *p* > 0.05). The highest indoor fungal aerosol concentration was observed at a physical exercise area of the Ważyn chamber (566 cfu/m^3^) and inside the Kołdras chamber (565 cfu/m^3^; Fig. 2b). At the same time, the highest outdoor fungal aerosol concentrations were up to 17-times higher than the indoor ones (Table 1). There were significant differences in fungal aerosol concentration in studied premises (with and without presence of patients) between fall and winter (Kruskal–Wallis test: *p* < 0.05; Fig. 3b).

During all bioaerosol measurements, environmental parameters, that is, temperature and relative humidity of the air, were also controlled. Median values and ranges for both these parameters measured indoors and outdoors are presented in Table 2. The collected data revealed high stability of thermal conditions. The temperature inside the studied premises ranged between 12 and 19 °C, and no significant differences were observed between them (Kruskal–Wallis test: *p* > 0.05). Despite the natural (in temperate climatic zone) fluctuations of outdoor air temperature over a year, there were also no differences between the median temperature values measured indoors and those noted in outdoor background.

**Table 2:** Temperature and relative humidity of the air at the Bochnia Salt Mine Health Resort

Environment	Temperature (°C)		Relative humidity (%)	
	Range	Median	Range	Median
Subterranean chambers				
During treatment courses with patients	15–19	17	39–73	61
After curative treatment without patients	13–19	16	25–71	55
Indoor background	12–19	16	38–70	64
Outdoor background	1–29	17	40–80	72

Compared to air temperature, the changes in relative humidity within a year in the health resort chambers were much more distinct. The humidity ranged from 25 % in winter to 73 % in summer and the median yearly value in the studied premises reached 56, and 70 % outdoors. Statistically significant differences in the examined premises were observed between summer (median: 70 %) and winter (median: 35 %) RH values only (Kruskal–Wallis test: *p* < 0.01).

The analyses of the influence of microclimatic parameters on the observed bacterial and fungal aerosol concentrations showed that only bacterial aerosol levels were affected by these factors. The correlation analysis revealed that each rise in temperature and relative humidity in the studied chambers resulted in significant increase in bacterial concentration in the air (Spearman correlation coefficient: *R* = 0.59 at *p* < 0.05 and *R* = 0.44 at *p* < 0.05, respectively). This relationship was the most significant in the Kołdras chamber (correlation coefficient: *R* = 0.75 at *p* < 0.05).

**Fig. 4:**
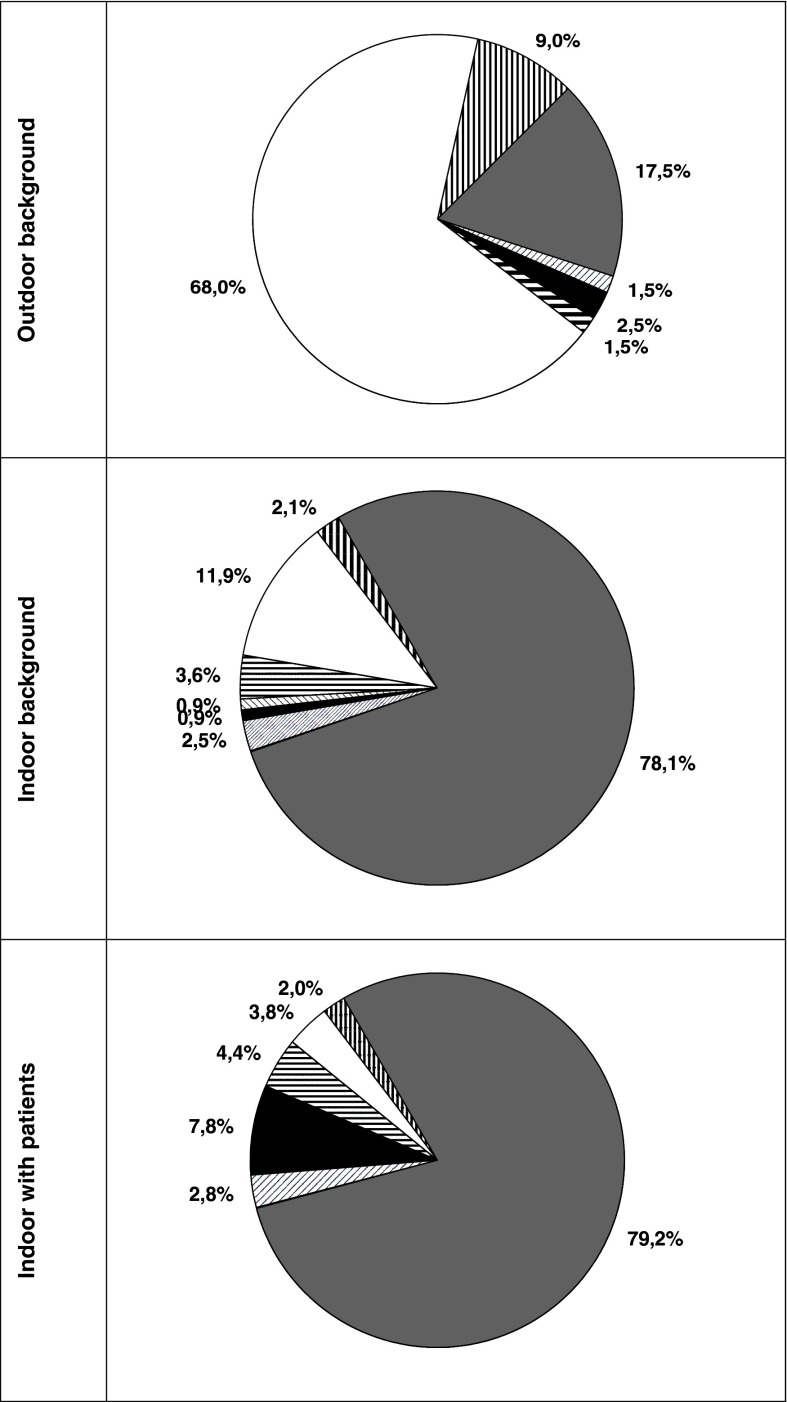
Percentage contribution (%) of bacterial and fungal groups to the total airborne microflora at the Bochnia Salt Mine Health Resort. The *symbols* represent:  Gram-positive cocci,  non-sporing Gram-positive rods,  endospore-forming Gram-positive rods,  Gram-negative rods,  mesophilic actinomycetes,  filamentous fungi and  yeasts

Figure 4 presents percentage contribution of bacterial and fungal groups to the total airborne microflora, and Table 3 specifies qualitatively these data showing the list of microbial taxa isolated from the air at the Bochnia Salt Mine Health Resort. The analysis of taxonomic composition of the air revealed that mesophilic Gram-positive cocci (*Micrococcus, Staphylococcus*), mesophilic actinomycetes (mainly *Streptomyces*) and molds (*Cladosporium* and *Penicillium*) predominated, which is a typical picture for both outdoor and indoor microflora. Molds (68 %), Gram-positive cocci (17.5 %) and yeast-like fungi (9 %) were the most prevalent in the studied outdoor environment. Gram-positive cocci were the most numerous among all analyzed microbial groups in the studied chambers. Irrespective to the presence of patients their contribution to the total bacterial community indoors reached 78–79 %. Endospore-forming Gram-positive rods were also frequently isolated from the air of studied premises. Their percentage contribution to the total airborne microflora was about 8 %. The presence of yeast-like fungi in the air was 2 %. Qualitative analyses revealed that the most numerous species belong to: among bacteria—*Staphylococcus* (8 species) and *Bacillus* (4 species) genera, and among fungi—*Penicillium* genus (4 species). The conditions inside the studied chambers favor the growth of *Staphylococcus* and *Bacillus* species. The bacteria from genus *Bacillus* were the most numerous in a bedroom in the Ważyn chamber (when the patients were present) but were also frequently isolated from the outdoor air. The air was free from Gram-negative rods in all analyzed subterranean chambers when the patients were present. It shows that there are unfavorable conditions for these micro-organisms survival and lack of any indoor sources of their emission and infiltration. The percentage contribution of Gram-positive cocci to the total number of bacteria in the studied chambers, when the patients were present, ranged from 70 to 96 %, and when the premises were empty—from 82 to 95 %. In this study, mesophilic Gram-positive cocci (mainly *Staphylococcus* and *Micrococcus*) predominated among identified micro-organisms of the subterranean chambers. Their percentage contribution to the total microflora ranged from 56 to 86 % and was significantly higher than those noted for outdoor background, where ranged from 24 to 29 % (Mann–Whitney test: *p* < 0.05). Frequent occurrence of *Staphylococcus* (mainly *S. cohnii* ssp*. cohnii, S. capitis, S. sciuri*) in the studied premises was noted. Their percentage contribution to the total bacterial microflora ranged from 8 to 27 %. In the examined premises, their highest concentration was particularly evident in the Kołdras chamber and in the physical activity area of the Ważyn chamber. Their high isolation frequency in the outdoor air (between 24 and 57 %) was also naturally reflected in their high abundance in the analyzed chambers (probably due to the transport through forced ventilation). Among isolated molds, *Cladosporium cladosporioides* predominated constituting from 42 to 66 % of the total fungal microflora in the studied chambers.

**Table 3:** Micro-organisms isolated from the air at the Bochnia Salt Mine Health Resort

Micro-organisms	Percentage contribution to the bacterial or fungal flora (%)	
	Indoor air	Outdoor air
Bacteria		
Gram-positive cocci		
*Kocuria rosea* ^a^	<1	8
*Microccocus* spp.	72	26
*Micrococcus luteus*	<1	8
*Staphylococcus epidermidis* ^a^	3	NI
*Staphylococcus sciuri* ^a^	3	4
*Staphylococcus lentus*	1	NI
*Staphylococcus hominis* ^a^	<1	NI
*Staphylococcus cohnii* ssp. *cohnii*	4	1
*Staphylococcus capitis*	2	10
*Staphylococcus haemolyticus*	<1	5
*Staphylococcus xylosus*	2	13
*Staphylococcus* spp.	1	NI
Non-sporing Gram-positive rods		
*Brevibacterium* spp.	1	5
*Corynebacterium* spp.	1	3
*Microbacterium* spp.^a^	<1	NI
Endospore-forming Gram-positive rods		
*Bacillus cereus* ^a^	1	10
*Bacillus licheniformis* ^a^	<1	2
*Bacillus polymyxa* ^a^	2	NI
*Bacillus pumilus*	<1	NI
*Bacillus* spp.	<1	NI
Gram-negative rods		
*Aeromonas hydrophila*	<1	NI
*Chryseobacterium indologenes*	<1	NI
Mesophilic actinomycetes		
*Nocardia* spp.	1	1
*Rhodococcus* spp.	1	2
*Streptomyces* spp.	2	2
Total bacteria	100	100
Fungi		
Filamentous fungi		
*Acremonium strictum*	2	7
*Acremonium* spp.	1	NI
*Alternaria alternata* ^a^	5	NI
*Alternaria chartarum*	1	NI
*Alternaria* spp.	2	1
*Aspergillus sydowii*	<1	NI
*Aspergillus terreus* ^a^	2	NI
*Aspergillus* spp.	NI	3
*Cladosporium cladosporioides*	29	NI
*Cladosporium herbarum*	NI	2
*Cladosporium macrocarpum*	<1	30
*Cladosporium* spp.	2	6
*Fusarium solani*	1	<1
*Fusarium* spp.	3	4
*Mucor plumbeus*	<1	<1
*Penicillium chrysogenum* ^a^	1	NI
*Penicillium crustosum* ^a^	2	NI
*Penicillium funiculosum*	5	NI
*Penicillium griseofulvum*	3	<1
*Penicillium* spp.	9	6
*Rhizopus stolonifer*	<1	<1
*Rhizopus* spp.	1	6
*Sporotrichum* spp.^a^	6	21
*Trichoderma viride* ^a^	3	NI
Yeasts		
*Candida* spp.	1	6
*Geotrichum candidum*	11	NI
*Geotrichum* spp.^a^	8	6
Total fungi	100	100

^a^Isolated only when the patients were present in the chambers

*NI* not isolated

## Discussion

Proper microbial quality of the air is an essential criterion to accept curative function of health resort and key factor in inhalation therapy (Frączek and Górny 2011; Prunk et al. 2008; Zhilina and Dobrodceeva 2005). Inhalation of naturally (including biological) and artificially formed aerosols may be advantageous for curative processes (Alkiewicz 1999; Amirav 2004; Anderson et al. 1997; Chervinskaya and Zilber 1995; Daviskas et al. 1996; Donaldson et al. 2006; Elkins et al. 2006). From medical point of view, specific curative conditions present in subterranean chambers or being created (sometimes artificially) in salt chambers have a beneficial effect on human health (Bihari-Axelsson and Axelsson 2002; Bis et al. 2004; Chervinskaya 2003, Chervinskaya and Zilber 1995, Fiegel et al. 2006 Frączek and Górny 2011). The quantitative analysis of bioaerosol in subterraneotherapy chambers at the Bochnia Salt Mine Health Resort revealed that indoor bacterial and fungal aerosol concentration were up to 1.2 × 10^4^ and 5.7 × 10^2^ cfu/m^3^, respectively. It shows that they were significantly higher than the concentrations reported by Dolezal et al. (1983) in subterranean chambers in Wieliczka Salt Mine.

The presence of patients in subterranean chambers had a significant effect (Mann–Whitney test: *p* < 0.01) on bacterial aerosol concentration both in each of the studied seasons and throughout the whole year. The highest bacterial aerosol concentration was observed during physical activity of the patients. The observed differences in bacterial aerosol concentrations between chambers result, interalia, from the fact that the Kołdras chamber is much smaller in terms of the size and bed capacity than the Ważyn chamber. The fact that the Ważyn chamber is adjusted for both the exercise therapy (sports field) and resting (sleeping area) is also important regarding its biological contamination.

Taking into account that the atmospheric air was pumped into all investigated chambers and the indoor bacterial aerosol concentration was significantly higher than outdoor, it is highly probable that the observed differences depended on the presence of patients and duration of their activity in studied chambers only. As it was shown, after several hours of patients’ presence, bacterial aerosol concentration in the chambers was significantly lower. These results suggest that the processes of self-purification of the air and/or gradual sedimentation of aerosols in calm indoor conditions are characteristic for these types of premises. A major role in this process is attributed to the presence of dry salt aerosols, which causes spore dehydration. A certain role in the self-attenuation of the subterranean premises from bioaerosol plays also the acid/alkaline relationship of the environment. Acidity of the environment in some subterranean chambers (sometimes even on the level of pH = 5) may have bacteriostatic properties (Olechnowicz-Bobrowska et al. 2000; Szczegielniak and Migała 2003). Similar relationship was detected by Grzyb et al. (2004) in subterranean chambers in Wieliczka. Such relationship was not observed for fungal aerosol, which is much more resistant to environmental stresses (such as desiccation, temperature changes, etc.) than bacterial one (Pastuszka 2001). The results showed that there were significant differences in fungal aerosol concentration in studied chambers irrespective to a presence or absence of the patients. Bis et al. (2004) investigating the air quality in subterranean chambers in Wieliczka Salt Mine observed similar relationship for molds. Quantitative analyze of fungal aerosol revealed that its concentration inside subterranean chambers (with presence and absence of patients) was significantly lower than in outdoor environment. It suggests that the atmospheric air can be the main source of fungal contamination of studied chambers and even mask an influence of the potential active indoor sources.

Górniok et al. (1977) stated that the quantitative presence of micro-organisms in subterranean chambers is several times lower than on the ground level. The relationships described in this study (regarding both fungi and bacteria) are comparable to the result obtained by other authors (Cox and Wathes 1995; Gąska-Jędruch and Dudzińska 2009; Lis et al. 1997; Wlazło et al. 2008). It is well known that the most important source of bacterial aerosol emission is humans. Their activity (including physiological functions like sneezing, coughing or intensive exhalation during physical exercises) is the main source of indoor bioaerosol (Frączek and Górny 2011; Gąska-Jędruch and Dudzińska 2009, Grzyb et al. 2004; Olechnowicz-Bobrowska et al. 2000). The obtained results suggest, however, that, apart from above mentioned factors, a proper ventilation system may effectively eliminate bioaerosol contamination (especially fungal one), providing appropriate air purity of the health resort chambers.

The obtained results of both indoor and outdoor measurements of bioaerosol concentrations were compared with the Polish proposals for threshold limit values (TLVs), which are 5 × 10^3^ cfu/m^3^ for both bacteria and fungi in indoor and outdoor environments (Górny 2010). In almost all investigated premises (apart from the physical activity area of the Ważyn chamber), the average values of bioaerosol concentrations were below the recommended TLVs. Bacterial aerosol concentration within physical activity area in the Ważyn chamber (11,688 cfu/m^3^ noted during sport exercises carried out by patients) was the only value that exceeded the admissible level. Hence, a high-performance method of microbial contamination reduction should be introduced in such premises to protect the patients, who have already suffered from respiratory diseases.

There were no significant differences in microbial aerosol concentrations between the studied seasons (Kruskal–Wallis test: *p* > 0.05) for indoor and outdoor backgrounds. This observation is different from those noted by the other authors in numerous “overground” locations where seasonal fluctuations in bioaerosol concentrations are quite typical (e.g., Górny and Dutkiewicz 2002; Jones and Harrison 2004; Lis et al. 1997; Nunes et al. 2005; Pastuszka et al. 2000; Reponen et al. 1992; Wlazło et al. 2008) and may confirm curative properties of these specific underground environment.

Significant seasonal differences inside health resort chambers in Bochnia were only noted between summer and winter for humidity values. Analyzing the influence of microclimatic conditions on bioaerosol, no statistically significant effect of these parameters on fungal concentration was observed in all studied seasons. It may suggest that the quantity of fungal microflora of salt mine chambers is very stable and even some natural phenomenon such as condensation and subsequent so-called underground rain (often present in summer in salt mines) promoting a fungal growth, has no significant influence (Araujo et al. 2008; Douwes et al. 2003; Wanner et al. 1993). For example, Olechnowicz-Bobrowska and Wojkowski (2004) showed that the stability of temperature, humidity, air velocity and atmospheric pressure is characteristic for salt mine chambers. They also observed that daily fluctuations of temperature and humidity in outdoor environment have only minimal influence on these parameters inside health resort chambers.

Qualitative composition of microflora isolated from the air of salt mine chambers was similar to this isolated in other subterranean health resort premises (Bis et al. 2004; Doleżal et al. 1983; Grzyb et al. 2004). It is known from the scientific literature that in vast majority of cases, Gram-positive cocci are more numerous in indoor environment than Gram-negative rods (Flannigan et al. 1991; Górny et al. 2005; Mancinelli and Shulls 1978; Niesler et al. 2010; Pastuszka et al. 2000). This regularity was also confirmed in salt mine chambers in Bochnia. Such numerous occurrences of the species from *Staphylococcus* genus are probably related to direct emission from the skin and respiratory tract (Dolezal et al. 1983; Gąska-Jędruch and Dudzińska 2009; Wlazło et al. 2008) as well as to their resistance to the relatively high content of NaCl in this environment. As it was shown, among bacteria, mesophilic Gram-positive cocci (mainly *Micrococcus* and *Staphylococcus*) prevailed also in the outdoor air (where isolation frequency ranged between 24 and 57 %). Hence, forced ventilation was also responsible for delivery of these microbial contaminants to the studied subterranean chambers. Fungal species from *Penicillium*, *Alternaria*, *Aspergillus* and *Cladosporium* genera were the most often isolated in salt mine chambers, and these results are in a good agreement with the observations conducted by other authors (e.g., Bis et al. 2004; Dolezal et al. 1983; Skrzyńska 1948). The results also indicate that aerobiological monitoring of health resort chambers should be periodically performed to confirm the proper indoor air quality necessary to maintain a curative function of these types of premises. It should be also pointed out that in this study, the microbiological contamination of both outdoor and indoor air including subterraneotherapy chambers was evaluated using impaction technique only and the final conclusion regarding the air quality was built based on the number of culturable bacteria and fungi. It is well known that bioaerosol sampling with impactors loaded with agar plates is used to separate and cultivate viable micro-organisms only. All of them can constitute up to 25 % of the total microbial particulates present in the environment, and the indoor quality evaluation based on the viable count only significantly underestimate the real exposure (Albrecht et al. 2007; Amann et al. 1995; Colwell and Grimes 2000; Flannigan 1999; Hanhela et al. 1995; Heikkila et al. 1988; Karlsson and Malmberg 1989; Kaeberlein et al. 2002; Sardessai 2005; Toivola et al. 2002). The latest results of the comparative analysis of viable and total concentrations of bacteria and fungi in the air showed that viable micro-organisms accounted for less than 3.9 % of the total airborne microflora (Gołofit-Szymczak and Górny 2010; Harkawy et al. 2011; Toivola et al. 2002). Hence, a control of the total number of micro-organisms (viable and non-viable together) provides a more precise characteristic of the air contamination than the measurements of viable bioaerosol concentrations only. It also allows predicting the level of exposure to harmful microbial agents in a more precise way (Gołofit-Szymczak 2012). According to Hung et al. (2005), although the sampling of viable micro-organisms in the air can provide valuable information, air sampling of any kind is not an unfailing means of determining the existence of microbial contamination and must be coupled with other detailed information. Air sampling and subsequent culture on agar media has several limitations including, damage of the propagules during aspiration, temporal and spatial variability, sampler performance and correctness of the taxa identification. Nevertheless, so far in everyday practice, the assessment of microbial air quality is limited to the measurement of viable bacterial and fungal particles, that is, single microbial cells, spores, their aggregates or fragments which are able to grow on proper agar medium as separate colonies. As the impaction technique and subsequent culture-based monitoring may underestimate the total exposure to bioaerosols (Górny et al. 2011; Niesler et al. 2010), other techniques that allow for identification of both culturable and non-culturable micro-organisms should be used to supplement standard methods in this type of studies in the future.

## Conclusions

The bioaerosol concentrations in subterraneotherapy chambers at the Bochnia Salt Mine Health Resort ranged from 35 to 11,688 cfu/m^3^ and from 189 to 2,517 cfu/m^3^ in indoor and outdoor air, respectively. The highest bioaerosol concentrations were observed (11,688 cfu/m^3^) when patients were present, and physical exercises were carried out in the chambers. These confirm that human and its different activities including physiological (sneezing, coughing, talking, etc.) and physical (e.g., therapeutic exercises) are the main source of bacterial aerosol in this indoor environment. The air contamination with fungal aerosol in investigated subterranean chambers was lower than outdoors (7–566 vs. 28–9,731 cfu/m^3^, respectively). The results clearly indicate that more attention should be paid to a quality of the air, which is delivered to subterraneotherapy chambers through the shafts and corridors. The microbial analyses of the air in indoor and outdoor environment revealed that Gram-positive cocci (*Micrococcus, Staphylococcus*), mesophilic actinomycetes (mainly *Streptomyces*) and molds (*Cladosporium* and *Penicillium*) were the predominant micro-organisms. As Gram-positive cocci can be easily released in high quantities from human body into the air, the number of people entering subterranean chambers should be limited. As the level of bacterial aerosol in subterranean chambers was significantly influenced with temperature and relative humidity, a constant control of these parameters should be scrupulously superintended in this type of premises dedicated for therapeutic purposes.
